# 

**Published:** 1897-06-26

**Authors:** 


**CADBURY'S COCOA**
"The standard of h ghest purity at present attainable."— *Lancet.*

**CADBURY'S COCOA**

NO ALKALIES USED.

### THE HOSPITAL

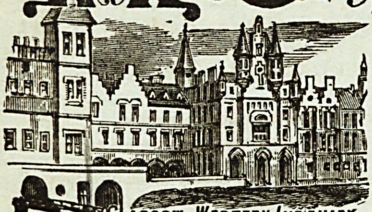

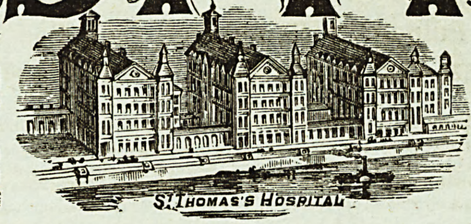

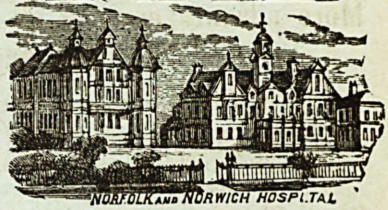

No. 561. Vol. XXII. [Registered as a Newspaper.] SATURDAY, JUNE 26TH, 1897. [Subscription Terms, see p. 210.] PRICE TWOPENCE.

